# Italian local chicken breeds: a comparative analysis of biodiversity on a global scale

**DOI:** 10.1186/s12711-025-00980-4

**Published:** 2025-06-10

**Authors:** Filippo Cendron, Christian Persichilli, Gabriele Senczuk, Francesco Perini, Emiliano Lasagna, Martino Cassandro, Mauro Penasa

**Affiliations:** 1 Department of Agronomy, Food, Natural Resources, Animals and Environment, University of Padova, 35020 Legnaro, Italy; 2 Department of Agricultural, Environmental and Food Sciences, University of Molise, 86100 Campobasso, Italy; 3 Department of Agricultural, Food and Environmental Sciences, University of Perugia, 06121 Perugia, Italy; 4 Federazione Delle Associazioni Nazionali Di Razza E Specie, 00187 Rome, Italy

## Abstract

**Background:**

Chickens, domesticated around 3500 years ago, are crucial in global agriculture, resulting in hundreds of breeds worldwide. In Europe, intensive breeding has led to the creation of numerous distinct commercial lines at the expense of local breeds. As a result, local breeds, which are not subject to rigorous selective practices, face higher risks of genetic problems due to a narrower genetic base. Modern genotyping and bioinformatic approaches allow detailed genetic analysis. This study offers a comprehensive genetic overview of Italian chicken biodiversity compared to global breeds, emphasizing the importance of preserving local genetic diversity.

**Results:**

Hundred and ninety-two chicken breeds from various countries were analyzed, with Italian breeds being highly represented. Genetic relationships showed that Italian breeds clustered with some European, African, and Asian breeds. The ADMIXTURE analysis identified 25 distinct populations and highlighted genetic similarities of certain Italian breeds with German, French, and Swiss ones. Genetic diversity was high in African and some Asian and European breeds, with Italian breeds exhibiting moderate diversity and variability. The TreeMix analysis revealed significant migration events and evolutionary clustering. The Italian breeds had close genetic ties and some highlighted evidence of genetic introgression from common ancestors.

**Conclusions:**

Italian chicken breeds have significant genetic relationships with European, Asian, and African breeds, reflecting historical trade and breeding exchanges. Southern Italian breeds form a distinct genetic group, highlighting regional uniqueness. Overall, the research points out the need for conservation strategies to preserve genetic diversity and account for historical and contemporary gene flows, ensuring the sustainability of Italian chicken biodiversity in the face of environmental and agricultural challenges.

**Supplementary Information:**

The online version contains supplementary material available at 10.1186/s12711-025-00980-4.

## Background

Chickens are one of the most extensively utilized species in livestock globally and the poultry industry currently produces over 120 million tons of meat and more than 1.2 trillion eggs annually [1]. Their domestication commenced ~ 3500 years ago [2], likely through a commensal pathway where initial interactions represented an adaptation of wild jungle fowl to niches created by humans. Subsequently, there was an increasing human intent to maintain, control, intensively breed, and ultimately commercialize chickens [3]. Throughout this process, hundreds of chicken breeds were developed worldwide, ranging from extensively raised indigenous breeds to highly specialized strains used in industrial-scale egg (layers) or meat (broilers) production [1]. Several domestication routes have been reported to lead to Europe, Africa, and South America [4, 5]. From Asia, chickens likely reached Europe through the Mediterranean region and northward through China and Russia to Northern Europe [6]. African chickens are thought to have descended from both European and Asian chicken strains [6, 7]. Despite the debate over whether South American chickens originated from Polynesian or European breeds [8], it is evident that both European and Asian breeding contributed to South American chicken breeds. Various local Asian and European breeds served as founding stocks for the development of commercial broilers and layers. Subsequently, commercial lines underwent intensive selection for production purposes, such as meat and egg production, as well as feed conversion efficiency [8].

In Europe, numerous chicken breeds have evolved as locally adapted populations, shaped by environmental conditions and management practices, as well as through selective breeding aimed at achieving desired phenotypic traits. While historical selection relied heavily on the phenotype of animals and breeder preferences rather than formal quantitative genetic approaches, these processes often involved a small number of founder animals, resulting in strong founder effects [9–11]. In the nineteenth century, as local chicken breeds that had been maintained for centuries gained popularity, the process of formal breed standardization began to take shape, especially with the establishment of poultry exhibitions and herdbooks in Europe. However, some breeds like the Silky and the Sicilian Buttercup were recognized and described much earlier. For example, the Sicilian Buttercup was associated with the royal family of Sicily in the twelfth century. At the same time, Asian breeds like Cochin and Langshan were introduced to Europe and crossbreeding between these breeds and local European populations led to the creation of new breeds, adapted to productivity goals and farming practices [12–14]. Current local chicken breeding in Europe is marked by limited exchange of breeding individuals, leading to population fragmentation that fosters inbreeding when population sizes are small. In contrast, in Asian, African, and South American countries, local chicken breeds are often raised by village residents using extensive farming systems with little or no selection and minimal exchange of breeding stock between neighboring villages [13–18]. Due to the often-low productivity of unselected local breeds in developing countries, the production of local breeds has been jeopardized by commercial breeding and the introduction of crosses to enhance productivity [8, 15, 19, 20]. Global genetic diversity of chickens has been explored, mostly thanks to projects such as SYNBREED, which have provided the opportunity to assess poultry biodiversity within the global poultry panorama over the past years [8].

In Italy, the interest in conserving local poultry breeds began several years ago with regional conservation plans, recently followed by national involvement [21, 22]. Within this context, the TuBAvI project and its continuation with the TuBAvI-2 project (https://www.pollitaliani.it/en/) have been developed to preserve, monitor, enhance, and valorize the genetic heritage of Italian poultry resources [23].

With the advent of accessible and high-throughput genotyping techniques, together with bioinformatic techniques to manage big data, it is now possible to conduct in-depth analyses of the genetic structure of chicken populations [8, 24]. These technologies have provided new insights into livestock genetics by introducing genomic approaches in conservation programs for small and endangered populations [25]. While some of the breeds included in the aforementioned projects have been analyzed using molecular markers [22, 26, 27], the lack of genome-wide association studies for these populations has limited the possibility of inferring the impact of genetic traits on whole-genome variation.

The primary objective of the present study was to provide a high-resolution genetic overview of the entire Italian chicken biodiversity in comparison to the global chicken heritage. This overall goal was pursued through several sub-objectives. First, we aimed to unravel the complexity of Italian chicken breeds by examining their genetic relationships with breeds from various countries. Second, we conducted a population study to better understand and unravel the similarities and divergences between Italian chicken breeds and those distributed worldwide, shedding light on their origins. Third, we evaluated biodiversity on a global scale, across different countries and continents, to highlight the critical role of conservation efforts in Italy in counteracting the genetic erosion of its local heritage.

## Methods

### Animals and genotyping

All animals within the Italian dataset were genotyped using the Affymetrix Axiom 600 K Chicken Genotyping Array, as detailed in Cendron et al. [24]. The raw dataset was integrated with genotypic data from various chicken breeds worldwide to explore the genetic relationships between Italian and global biodiversity. Genotypic information for 3047 animals representing 167 breeds was obtained from Malomane et al. [8], establishing a global dataset. All individuals retrieved from Malomane et al. [8] were genotyped with the Affymetrix Axiom 600 K Chicken Genotyping Array. After merging, the final dataset consisted of 3720 animals, 467,723 common SNPs, and 192 breeds, divided according to geographical origin (Tables 1 and 2). The data were edited using the PLINK 1.9 software [28] to remove SNPs with a call rate lower than 95%, SNPs with a minor allele frequency lower than 5%, and animals with more than 10% missing genotypes. To avoid multicollinearity effects, the genotype data were subjected to linkage disequilibrium (LD) pruning using PLINK 1.9 [28], with a SNP window size of 50, a step size of 5 SNPs, and R^2^ of 0.60. After editing, genotypes of 673 animals from 25 Italian local chicken breeds and 467,723 SNPs were retained for statistical investigation.

**Table 1:** Number of breeds and animals by continent and country

Continent	Country	Number of breeds	Number of animals
Africa	Egypt	2	36
Africa	Ethiopia	2	59
Africa	Sudan	1	15
Africa	Tanzania	5	92
Africa	Zimbabwe	1	20
America	Argentina	1	19
America	Chile	3	59
America	USA	11	219
Asia	Bangladesh	1	30
Asia	China	11	217
Asia	Indian	1	20
Asia	Indonesia	2	33
Asia	Israel	1	20
Asia	Japan	11	223
Asia	Malaysia	2	30
Asia	Pacific	1	4
Asia	Pakistan	2	21
Asia	Philippines	1	34
Asia	Russia	2	43
Asia	Saudi Arabia	8	92
Asia	Thailand	2	38
Asia	Vietnam	8	151
Europe	Albania	1	19
Europe	Austria	1	18
Europe	Belgium	1	20
Europe	England	13	236
Europe	Finland	7	95
Europe	France	3	88
Europe	Germany	23	424
Europe	Hungary	2	40
Europe	Iceland	1	20
Europe	Italy	29	673
Europe	Norway	1	20
Europe	Poland	1	20
Europe	Spain	4	79
Europe	Switzerland	4	77
Europe	The Netherlands	3	45
Europe	Turkey	2	40
Europe	Ukraine	1	19
Commercial	Commercial	16	312

**Table 2:** General information on the breeds included in the study

Acronym	Breed name	Continent	Country	Project
708	Ross708	Commercial	Commercial	SYNBREED
ABwa	Antwerpener Bartzwerge, wachtelfarbig^a^	Europe	Belgium	SYNBREED
ACxx	Ac^a^	Asia	Vietnam	SYNBREED
AKxx	Altenglische Kämpfer, verschiedene Farben^a^	Europe	Austria	SYNBREED
ALHxx	ALH^a^	Europe	Finland	SYNBREED
ALxx	Albanian Crowers^a^	Europe	Albania	SYNBREED
ANC	Ancona	Europe	Italy	TUBAVI
ANxx	Aseel Pakistan^a^	Asia	Pakistan	SYNBREED
APsscht	Appenzeller Spitzhaube, silber-schwarz getupft^a^	Europe	Switzerland	SYNBREED
APxx	Appenzeller Spitzhaube^a^	Europe	Switzerland	SYNBREED
ARsch	Araucanas, schwarz^a^	Europe	England	SYNBREED
ARw	Araucanas, weiß^a^	Europe	England	SYNBREED
ARwi	Araucanas, wildfarbig^a^	Europe	England	SYNBREED
ASrb	Asil, rotbunt^a^	Asia	Indian	SYNBREED
AZxx	Appenzeller Barthuhn^a^	Europe	Switzerland	SYNBREED
BANG	Naked Neck^a^	Asia	Bangladesh	SYNBREED
BAsch	Bantam, schwarz^a^	Europe	Germany	SYNBREED
BBxx	Brabanter, verschiedene Farben^a^	Europe	The Netherlands	SYNBREED
BHrg	Brahma, rebhuhnfarbig^a^	America	USA	SYNBREED
BHwsch	Brahma, weiß-schwarzcolumbia^a^	America	USA	SYNBREED
BIxx	Bedouin^a^	Asia	Israel	SYNBREED
BKschg	Bergische Kräher, schwarz-goldbraungedobbelt^a^	Europe	Germany	SYNBREED
BL_A	Rhodeländer^a^	Commercial	Commercial	SYNBREED
BL_B	Rhodeländer^a^	Commercial	Commercial	SYNBREED
BL_C	Weiße Plymouth Rock^a^	Commercial	Commercial	SYNBREED
BL_D	Weiße Plymouth Rock^a^	Commercial	Commercial	SYNBREED
BLxx	Brakel, verschiedene Farben^a^	Europe	Germany	SYNBREED
BPT	Bionda Piemontese	Europe	Italy	TUBAVI
BRD_A	Broiler dam line^a^	Commercial	Commercial	SYNBREED
BRD_B	Broiler dam line^a^	Commercial	Commercial	SYNBREED
BRS_A	Broiler sire line^a^	Commercial	Commercial	SYNBREED
BRS_B	Broiler sire line^a^	Commercial	Commercial	SYNBREED
BRxx	Baier chicken^a^	Asia	China	SYNBREED
BSA	Bianca di Saluzzo	Europe	Italy	TUBAVI
BSsch	Bergische Schlotterkämme, schwarz^a^	Europe	Germany	SYNBREED
BUxx	Sicilian Buttercup^a^	Europe	Italy	SYNBREED
CAxx	Chahua chicken^a^	Asia	China	SYNBREED
CHgesch	Chabo, gelb mit schwarzen Schwanz^a^	Asia	Japan	SYNBREED
CHschw	Chabo, schwarz mit weißen Tupfen^a^	Asia	Japan	SYNBREED
CHxx	Chabo, verschiedene Farben^a^	Asia	Japan	SYNBREED
CIxx	Choi^a^	Asia	Vietnam	SYNBREED
CMsch	Cemani, schwarz^a^	Asia	Indonesia	SYNBREED
COR	Cornuta di Caltanissetta	Europe	Italy	TUBAVI
COsch	Cochin, schwarz^a^	Asia	China	SYNBREED
CWxx	Ching’wekwe^a^	Africa	Tanzania	SYNBREED
DAN	Dandarawi^a^	Africa	Egypt	SYNBREED
Desi	Desi Pakistan^a^	Asia	Pakistan	SYNBREED
DKschs	Denizlikräher, schwarz-silber^a^	Europe	Germany	SYNBREED
DKxx	Denizli^a^	Europe	Turkey	SYNBREED
DLla	Deutsche Lachshühner, lachsfarbig^a^	Europe	Germany	SYNBREED
DOUxx	Dou (Henan game)^a^	Asia	China	SYNBREED
DOxx	Dorking, verschiedene Farben^a^	Europe	England	SYNBREED
DSgp	Deutsche Sperber^a^	Europe	Germany	SYNBREED
DTxx	Dong Tao^a^	Asia	Vietnam	SYNBREED
DZgh	Deutsche Zwerghühner, goldhalsig^a^	Europe	Germany	SYNBREED
EUK	Eureka	Commercial	Commercial	SYNBREED
EUxx	Eulenbarthühner, verschiedene Farben^a^	Europe	Germany	SYNBREED
FAY	Fayoumi^a^	Africa	Egypt	SYNBREED
FINxx	TYR^a^	Europe	Finland	SYNBREED
FRgew	Friesenhuhn, gelb-weißgeflockt^a^	Europe	Germany	SYNBREED
FZgpo	Federfüßige Zwerghühner, gold-porzellanfarbig^a^	Europe	Germany	SYNBREED
FZsch	Federfüßige Zwerghühner, schwarz^a^	Europe	The Netherlands	SYNBREED
GBxx	Grübbe Bartzwerge, verschiedene Farben^a^	Europe	England	SYNBREED
GGg	Gallus Gallus Gallus^a^	Asia	Thailand	SYNBREED
GGsc	Gallus Gallus Spadiceus^a^	Asia	Thailand	SYNBREED
GRxx	Green legged Partridge^a^	Europe	Poland	SYNBREED
GUxx	Gushi chicken^a^	Asia	China	SYNBREED
GZxx	Gerze^a^	Europe	Turkey	SYNBREED
HAsl	Hamburger, silberlack^a^	Europe	Germany	SYNBREED
HORxx	HOR^a^	Europe	Finland	SYNBREED
HOxx	Holländer Weißhauben, verschiedene Farben^a^	Europe	The Netherlands	SYNBREED
HUschw	Houdan, schwarz-weißgescheckt^a^	Europe	France	SYNBREED
HUxx	Houdan^a^	Europe	France	SYNBREED
Hxx	Ho^a^	Asia	Vietnam	SYNBREED
HYL	Hyla	Commercial	Commercial	SYNBREED
IKxx	Indische Kämpfer, verschiedenen Farben^a^	Europe	England	SYNBREED
ILMxx	ILM^a^	Europe	Finland	SYNBREED
ILxx	Icelandic Landrace^a^	Europe	Iceland	SYNBREED
ISA	Isa Brown	Commercial	Commercial	SYNBREED
ITrh	Italiener, rebhuhnhalsig^a^	Europe	Italy	SYNBREED
ITsch	Italiener, schwarz^a^	Europe	Italy	SYNBREED
JAExx	Jaerhoens^a^	Europe	Norway	SYNBREED
KAsch	Kastilianer, schwarz^a^	Europe	Spain	SYNBREED
KIUxx	KIU^a^	Europe	Finland	SYNBREED
KRsch	Krüper, schwarz^a^	Europe	Germany	SYNBREED
KRw	Krüper, weiß^a^	Europe	Germany	SYNBREED
KRxx	Krüper, verschiedene Farben^a^	Europe	Germany	SYNBREED
KSgw	Ko Shamo, gold-weizenfarbig^a^	Asia	Japan	SYNBREED
KUxx	Kuchi^a^	Africa	Tanzania	SYNBREED
KYswi	Koeyoshi^a^	Asia	Japan	SYNBREED
LAco	Lakenfelder^a^	America	USA	SYNBREED
LER11	White Leghorn^a^	America	USA	SYNBREED
LEw	White Leghorn^a^	Europe	Germany	SYNBREED
LSxx	Langshan chicken^a^	Asia	China	SYNBREED
MAgw	Malaien,gold-weizenfarbig^a^	Asia	Malaysia	SYNBREED
MAPar	Mapuche^a^	America	Argentina	SYNBREED
MAPbio	Mapuche^a^	America	Chile	SYNBREED
MAPrio	Mapuche^a^	America	Chile	SYNBREED
MAPxx	Mapuche^a^	America	Chile	SYNBREED
MAxx	Malaien, verschiedene Farben^a^	Asia	Malaysia	SYNBREED
MER	Mericanel della Brianza	Europe	Italy	TUBAVI
MIAxx	Mia^a^	Asia	Vietnam	SYNBREED
MIsch	Minorka, schwarz^a^	Europe	Spain	SYNBREED
MOD	Modenese	Europe	Italy	TUBAVI
MOxx	Morogoro Medium^a^	Africa	Tanzania	SYNBREED
MRschk	Marans, schwarz-kupfer^a^	Europe	France	SYNBREED
MUG	Mugellese	Europe	Italy	TUBAVI
NHbr	New Hampshire, gold- braun^a^	America	USA	SYNBREED
NHL68	New Hampshire, gold-braun^a^	America	USA	SYNBREED
ODxx	Onaga dori, verschiedene Farben^a^	Asia	Japan	SYNBREED
OFrbx	Orloff, rotbunt^a^	Asia	Russia	SYNBREED
OHgh	Ohiki, goldhalsig^a^	Asia	Japan	SYNBREED
OHsh	Ohiki, silberhalsig^a^	Asia	Japan	SYNBREED
OMsschg	Ostfriesische Möwen, silber-schwarzgeflockt^a^	Europe	Germany	SYNBREED
ORge	Orpington, gelb^a^	Europe	England	SYNBREED
PAxx	Paduaner, verschiedene Farben^a^	Europe	Italy	SYNBREED
PCI	Collo Nudo Italiana	Europe	Italy	TUBAVI-2
PCxx	Malakula^a^	Asia	Pacific	SYNBREED
PER	Ermellinata di Rovigo	Europe	Italy	TUBAVI
PExx	Pemba^a^	Africa	Tanzania	SYNBREED
PHxx	Phoenix, verschiedene Farben^a^	America	USA	SYNBREED
PIIxx	PII^a^	Europe	Finland	SYNBREED
PIxx	Kibawe Bukidnon^a^	Asia	Philippines	SYNBREED
PLB	Livorno Bianca	Europe	Italy	TUBAVI
PLN	Livorno Nera	Europe	Italy	TUBAVI
PML	Millefiori di Lonigo	Europe	Italy	TUBAVI
PMP	Millefiori Piemontese	Europe	Italy	TUBAVI-2
PPA	Padovana Argentata	Europe	Italy	TUBAVI
PPB	Polverara Bianca	Europe	Italy	TUBAVI
PPC	Padovana Camosciata	Europe	Italy	TUBAVI
PPD	Padovana Dorata	Europe	Italy	TUBAVI
PPN	Polverara Nera	Europe	Italy	TUBAVI
PPP	Pepoi	Europe	Italy	TUBAVI
PRgp	Plymouth Rock, gestreift^a^	America	USA	SYNBREED
PRL	Robusta Lionata	Europe	Italy	TUBAVI
PRM	Robusta Maculata	Europe	Italy	TUBAVI
PTxx	Prathuhn^a^	Europe	Spain	SYNBREED
RHrh	Rheinländer, rebhuhnhalsig^a^	Europe	Germany	SYNBREED
RHsch	Rheinländer, schwarz^a^	Europe	Germany	SYNBREED
RIxx	Ri^a^	Asia	Vietnam	SYNBREED
ROM	Romagnola	Europe	Italy	TUBAVI
ROro	Rhodeländer, dunkelrot^a^	America	USA	SYNBREED
RVxx	Red Villafranquina^a^	Europe	Spain	SYNBREED
SAsch	Sumatra, schwarz^a^	Asia	Indonesia	SYNBREED
SAU10	Saudi10^a^	Asia	Saudi Arabia	SYNBREED
SAU3	Saudi3^a^	Asia	Saudi Arabia	SYNBREED
SAU4	Saudi4^a^	Asia	Saudi Arabia	SYNBREED
SAU5	Saudi5^a^	Asia	Saudi Arabia	SYNBREED
SAU6	Saudi6^a^	Asia	Saudi Arabia	SYNBREED
SAU7	Saudi7^a^	Asia	Saudi Arabia	SYNBREED
SAU8	Saudi8^a^	Asia	Saudi Arabia	SYNBREED
SAU9	Saudi9^a^	Asia	Saudi Arabia	SYNBREED
SAVxx	SAV^a^	Europe	Finland	SYNBREED
SBgschs	Sebright, gold^a^	Europe	England	SYNBREED
SBsschs	Sebright, silber^a^	Europe	England	SYNBREED
SCw	Schweizer Huhn^a^	Europe	Switzerland	SYNBREED
SEsch	Seidenhühner, schwarz^a^	Asia	China	SYNBREED
SEw	Seidenhühner, weiß^a^	Asia	China	SYNBREED
SHsch	Shamo, schwarz^a^	Asia	Japan	SYNBREED
SIC	Siciliana	Europe	Italy	TUBAVI
SNwsch	Sundheimer, weiß-schwarzcolumbia^a^	Europe	England	SYNBREED
SUDxx	Large Beladi^a^	Africa	Sudan	SYNBREED
SUw	Sultanhühner, weiß^a^	Europe	England	SYNBREED
TakH	Horro^a^	Africa	Ethiopia	SYNBREED
TakJ	Jarso^a^	Africa	Ethiopia	SYNBREED
TExx	Te^a^	Asia	Vietnam	SYNBREED
THsch	Thüringer Barthühner, schwarz^a^	Europe	Germany	SYNBREED
TNN	Transilvanien Naked Neck^a^	Europe	Hungary	SYNBREED
TOgh	Totenko, goldhalsig^a^	Asia	Japan	SYNBREED
TVxx	Tau Vang^a^	Asia	Vietnam	SYNBREED
UBxx	Ukrainian bearded^a^	Europe	Ukraine	SYNBREED
UNxx	Unguja^a^	Africa	Tanzania	SYNBREED
VLD	Valdarnese	Europe	Italy	TUBAVI
VLP	Valplatani	Europe	Italy	TUBAVI
VWco	Vorwerkhuhn^a^	Europe	Germany	SYNBREED
VWcoE	Vorwerkhuhn^a^	Europe	Germany	SYNBREED
WDxx	Wannan Three yellow^a^	Asia	China	SYNBREED
WL_A	Weiße Leghorn^a^	Commercial	Commercial	SYNBREED
WL_B	Weiße Leghorn^a^	Commercial	Commercial	SYNBREED
WL_C	Weiße Leghorn^a^	Commercial	Commercial	SYNBREED
WL_D	Weiße Leghorn^a^	Commercial	Commercial	SYNBREED
WTs	Westfälische Totleger, silber^a^	Europe	Germany	SYNBREED
WUxx	Wugu^a^	Asia	China	SYNBREED
WYsschs	Wyandotten, silber schwarzgesäumt^a^	America	USA	SYNBREED
WYw	Wyandotten, weiß^a^	America	USA	SYNBREED
XSxx	Xiaoshan chicken^a^	Asia	China	SYNBREED
YH	Hungarian Yellow^a^	Europe	Hungary	SYNBREED
YKxx	Yurlov Crower^a^	Asia	Russia	SYNBREED
YOwr	Yokohama, weiß-rot gezeicnet^a^	Asia	Japan	SYNBREED
ZCsch	Zwerg Cochin, schwarz^a^	Europe	England	SYNBREED
ZCw	Zwerg Cochin, weiß^a^	Europe	England	SYNBREED
ZIMxx	Ecotype^a^	Africa	Zimbabwe	SYNBREED
*Names from Malomane et al.				

The Gallus_gallus-6.0 chicken assembly (GRCg6a) was used as the reference genome [24], with markers positioned on chromosomes 1 to 28. The GRCg6a assembly was used as it was built on Red Jungle Fowl, which is similar to the local breeds included here, rather than the GCRg7b assembly, a more updated reference genome but built on broiler genomic features [29].

### Multidimensional scaling and phylogenetic analysis

Pair-wise genetic relationships within and between the breeds were estimated using a matrix of genome-wide identity-by-state genetic distances in PLINK 1.9 [28] and plotted using a multidimensional scaling (MDS) plot that represented components C1 and C2. Phylogenetic relationships among the breeds were assessed using Reynold's genetic distances in the RStudio package ‘ape’ [30]. A neighbor-joining tree was constructed based on individual allele-sharing distances (-distance 1-IBS in PLINK) and visualized using FigTree [30, 31].

### Admixture and genetic relationships

Population structure was investigated by applying the model-based clustering algorithm in the ADMIXTURE software, with the number of ancestral populations (K) ranging from 2 to 35 [32]. The cross-validation procedure was used to determine the most likely number of ancestral populations as the value of K that minimized the cross-validation prediction error. The BITE R package was used to graphically represent the results through the membercoef.circos function [33].

### Genetic diversity indices

The PLINK 1.9 software [28] was used to compute observed (H_O_) and expected heterozygosity levels (H_E_), as well as the inbreeding coefficient, which was based on excess of homozygosity (F_HOM_) [34]. Identification of runs of homozigosity (ROH) involved the following parameters: (i) minimum length of 1 Mb (–homozig-kb); (ii) allowance of two missing SNPs and up to one heterozygous genotype within the ROH (-homozyg-window-missing 50 and –homozyg-window-het 1); (iii) at least 50 SNPs included (-homozyg-snp 50); (iv) minimum SNP density in the ROH of at least one SNP per 100 kb (-homozyg-density 100); and (v) maximum gap of 1000 kb between consecutive homozygous SNPs (-homozyg-gap 1000). The genomic inbreeding coefficient using the ROH data (F_ROH_) of an individual was computed as total length of the genome covered by ROH divided by the total length of the autosomal genome length that was covered by the SNP array [35].

### Population structure

To infer patterns of population splitting and introgression events within the phylogenetic framework of Italian breeds, the software TreeMixv1.13 [36], which implements a maximum likelihood approach based on allele frequencies, was utilized with the bootstrap procedure.

On the basis of the observed overall genetic structure and to have a more resolute investigation on Italian poultry breeds, a subset composed of Italian populations, four commercial White Leghorn (WL) lines that are known for their similarity to the local breeds PLB and PLN, and two wild breeds (Gallus gallus gallus and Gallus gallus spadeaceus) was created. Thus, the Ggg genotypes were used as an outgroup. The decision to limit the TreeMix analysis to these breeds was also due to the absence of prior analyses on local Italian chicken breeds and to the difficulty of performing the analysis on all breeds due to the substantial computational burden required. The pipeline consists of three main steps. First, a preliminary run of TreeMixv1.13 was performed with 10 replicates, assuming migration edges ranging from 0 to 10. During this step, using the results of TreeMix, a consensus tree was built using the software PHYLIP v3.6 [37]. In the second step, the optimal number of migration edges was determined using the linear method implemented in the optm function of the OptM R package [38]. Finally, following 15 distinct iterations, a consensus tree was constructed using the most supported number of migrations, as previously estimated. To determine the robustness of the nodes of underlying Treemix graph, the initial and final phases were performed using 1000 bootstraps, while accounting for LD within blocks of 500 SNPs.

## Results

### Breeds and samples

Table 1 summarizes the number of breeds and animals for each country within the entire global heritage. A total of 192 breeds from 39 countries were compared, alongside commercial lines (Table 2). The largest number of breeds and animals was observed in Italy, due to the availability of a comprehensive dataset that was collected in the TuBAvI and TuBAvI-2 projects. The remaining populations accounted for 14 to 30 animals per breed. It is worth reporting that certain breeds were considered based on the respective country where they are typically found, rather than the physical location where they were sampled. For instance, the Padovana breed included in the dataset of Malomane et al. [8] and was assigned to the Italian poultry heritage, prioritising its geographical origin than considering whether it was raised or sampled in countries outside Italy, such as Germany. Also, the ITrh, ITsch, Buxx, and Paxx breeds were originally not included in our starting dataset but were included in the dataset of Malomane et al. [8], where they are classified as Italian.

### Genetic relationships

**Fig. 1:**
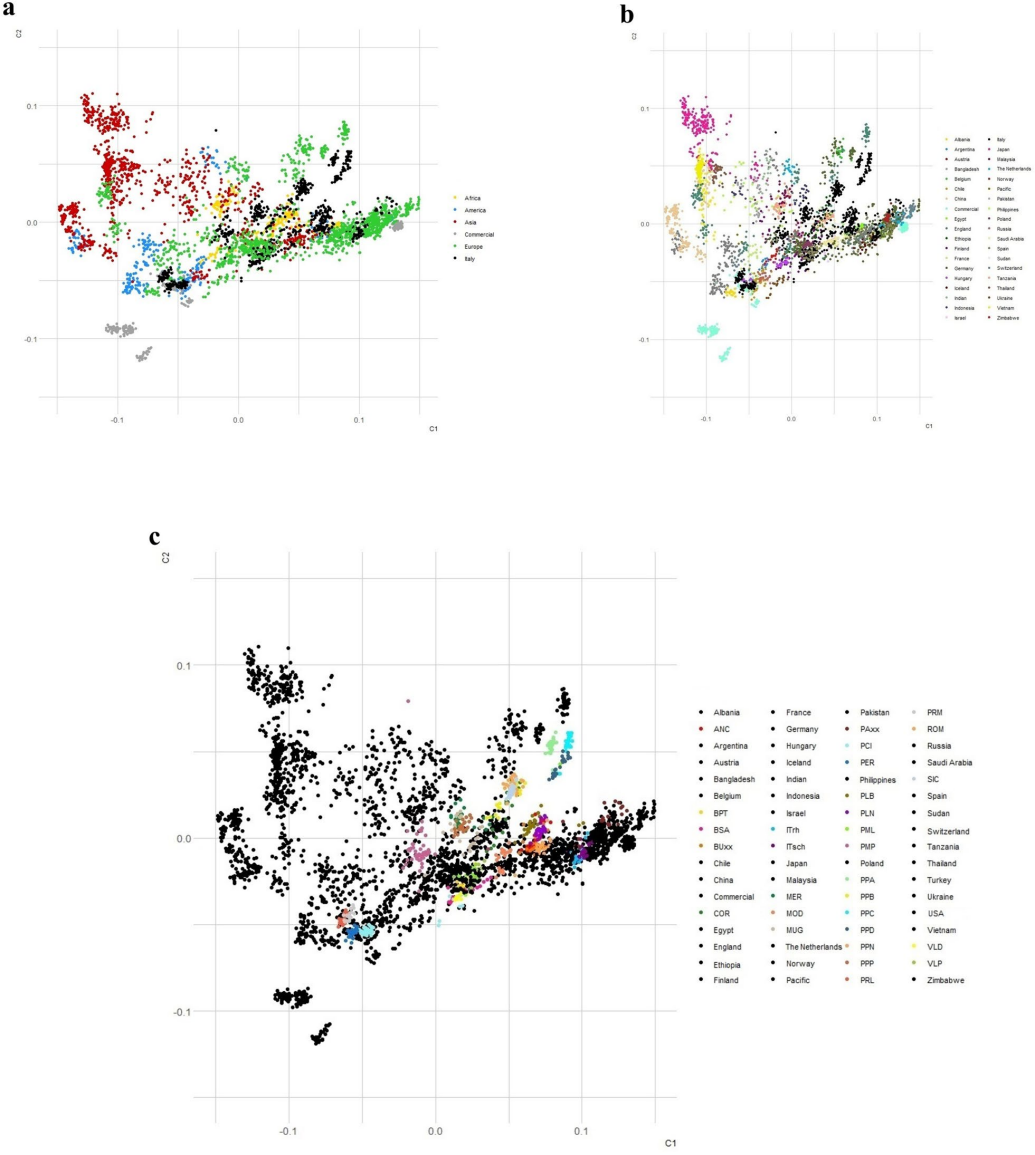
Genetic relationships among the 192 chicken breeds, as inferred by multidimensional scaling analysis. **a** Comparison of Italian breeds with populations based on their continental distribution, **b** clustering of breeds by nationality, and **c** distribution of individual local Italian breeds compared to breeds from their respective countries of origin. Breed acronyms are in Table 2

Results of the MDS analysis presented in Fig. 1 elucidate the relationships among various breeds. Initially, as the focus was on evaluating Italian breeds within the global chicken heritage, we segregated Italian breeds from the rest of the dataset to assess their closest affiliations at the continental level. The Italian breeds were found to share genetic relationships with the majority of breeds from Europe, Africa, and, to a lesser extent, Asia, with a distinct isolated cluster that overlapped with commercial lines (Fig. 1a). Figure 1b provides a higher resolution of these results by zooming in to understand the genetic relationships among Italian breeds at country level. Notably, some Italian breeds were genetically connected with Austrian, Spanish, and German breeds. Furthermore, certain Italian breeds clustered with breeds from Malaysia, Egypt, and Israel. The data dispersion in the central part of the MDS suggests that most European and African breeds in this study were closely related genetically, while those from Asia and America followed distinct clustering patterns.

To gain a more insight into the genetic proximity of Italian breeds with global counterparts, Fig. 1c depicts the MDS plot for each Italian breed. Italian breeds that overlapped with commercial lines included PRL, PER, PCI, PRM, and ITrh. Additionally, breeds associated with the European poultry heritage included key Italian breeds such as PPD, PPC, PPA, PPN, and PPB. Interestingly, breeds from southern Italy such as SIC and BUxx, aligned with the cluster of breeds from African countries, such as Egypt.

**Fig. 2:**
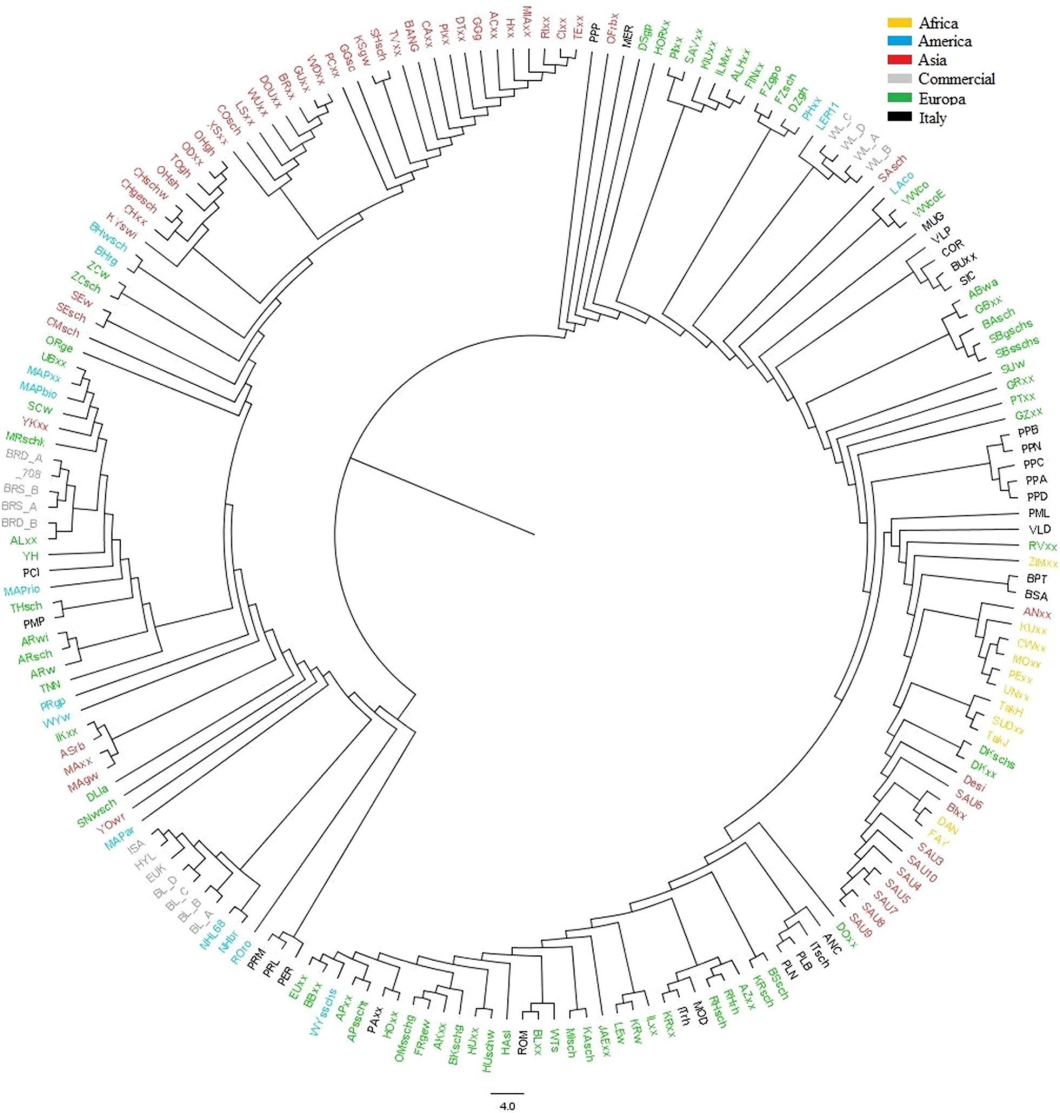
Neighbor-joining tree based on Reynold’s genetic distances for the breeds. Breed acronyms are in Table 2

Figure 2 depicts the phylogenetic tree obtained using Reynold’s genetic distances. From a general perspective, this analysis revealed that certain breeds tended to cluster according to their continent of origin, indicating a notable pattern of genetic diffusion within their respective geographical regions. In the upper left part of Fig. 2, a strong proximity among various Asian breeds was evident, while in the lower right part, a cluster was identified that comprises African breeds, partly related to Asian breeds. Breeds belonging to the European region formed three distinct clusters. Notably, the distribution of Italian breeds intertwined with various breeds worldwide, indicating proximity to breeds from multiple countries. It is interesting to note how the PCI breed closely aligned with commercial lines and the Hungarian YH breed, originating from the Hungarian TNN, while PPP and MER were close to the German DSgp breed. The PCI and TNN breeds did not share the same haplotype in the chromosomal region associated with the naked neck phenotype, which may explain why PCI was more similar to YH, likely attributable to the crossbreeding that has occurred over time (see Additional file 1: Figure S1).

Southern Italian breeds VLP, COR, Buxx, and SIC were strongly associated, forming a distinct branch in the phylogenetic tree. Similarly, breeds PPA, PPC, PPD, PPN, and PPB created a separate branch in the phylogenetic tree, somewhat connected to the Turkish-origin GZxx. The Italian breeds PRM, PRL, and PER had a clear phylogenetic proximity, somewhat associable with commercial layer-type breeds and the United States breeds ROro, NHji, and NHL68.

### Genetic structure

**Fig. 3:**
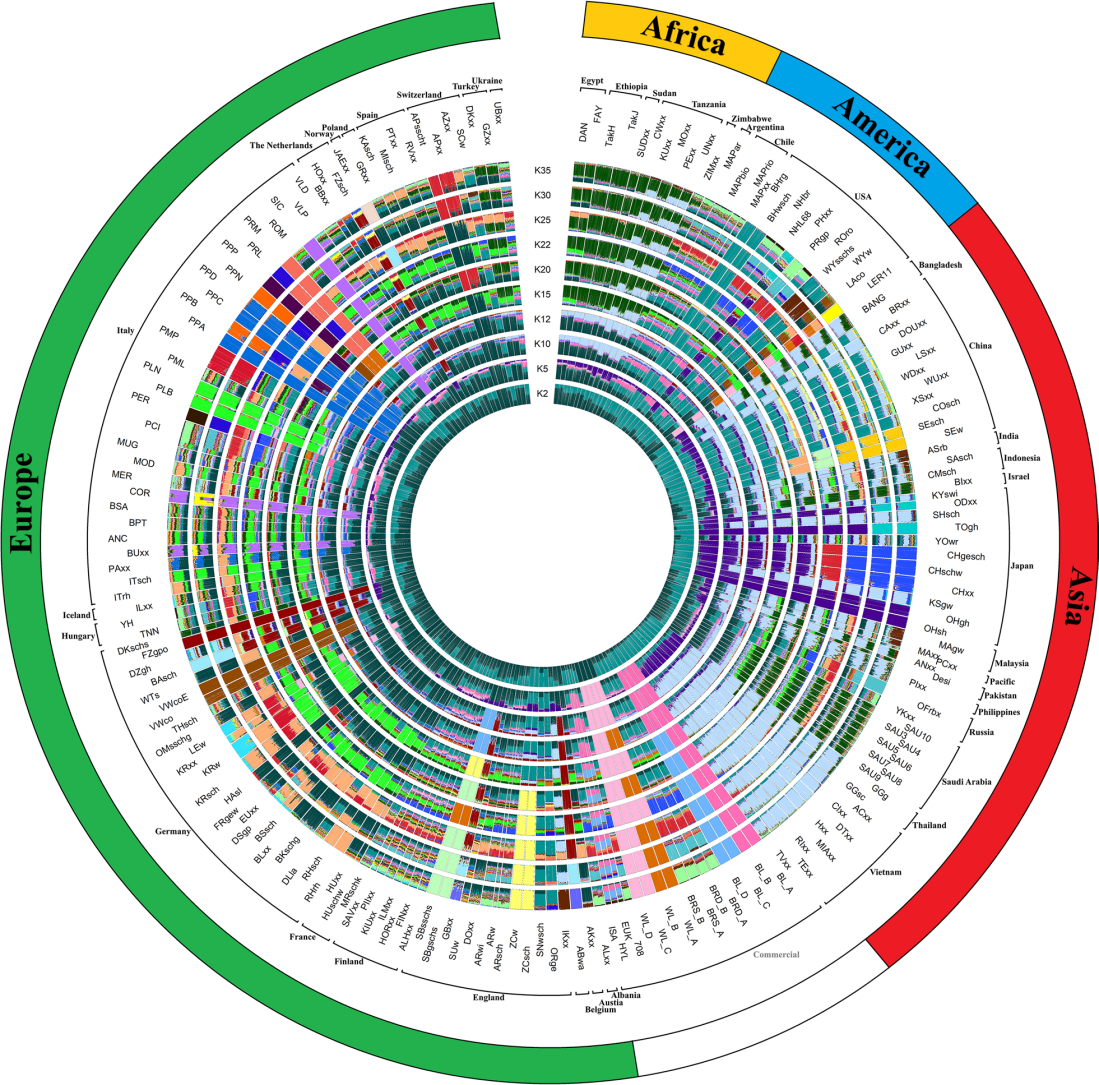
Population structure of the 192 chicken breeds as revealed by ADMIXTURE analysis across K = 2 to K = 35 clusters. The circular plot illustrates the genetic ancestry proportions of each breed, organized by continent and country of origin. Breed acronyms are in Table 2

The population structure obtained from the ADMIXTURE analysis (Fig. 3) using from 2 to 35 potential clusters (K), indicated that the most suitable number of populations within the total sample was K = 25. European breeds distinctly separated from those of other continents at each represented K. At K = 2, two overarching genetic backgrounds were discernible, allowing for the subdivision of European breeds from those from other continents. At K = 22, the Italian PLN, PLB, and MOD breeds had genomic background similar to German (LEw, KRxx, KRw, KRsch, DSgp, BSsch, BLxx, BKschg, and DLIA) and French breeds (HUxx, RHsch, and Rhrh). At K = 35, the PMP breed had a genetic background almost entirely identical to the Swiss chicken breeds APsscht and APxx. Although Italian breeds were genetically closer to commercial lines based on the MDS (Fig. 1), no marked genetic proximity with commercial lines was observed. The SIC and Buxx breeds, which belong to the same population but were sampled and raised in different locations (Italy and Germany, respectively), exhibited the same genetic background. However, the BUxx breed had structural genetic variants that suggest impurity compared to the local SIC breed of our dataset. For the Italian PCI breed, subgroups within the population were noticeable at K = 12 and K = 15, indicating differences in the genetic background within this breed. At K = 25,Japanese breeds such as CHgesch, CHschw, and CHxx had genetic proximity to Italian PPC, PPD, and PPA breeds, which was not supported by the phylogenetic tree (Fig. 2).

Commercial chicken lines included in our dataset, i.e., EUK, HYL, and ISA, did not show genetic analogy with other commercial lines that were retrieved from the dataset of Malomane et al. [8]. On the other hand, the ROSS 708 from our dataset strongly resembled commercial broilers BRS_A, BRS_B, BRD_A, and BRD_B.

Breeds from Saudi Arabia (SAU) had a distinctly shared genetic background with breeds from Egypt (DAN, FAY), Ethiopia (TakH, TakJ), and Sudan (SUDxx) (Fig. 3). Additionally, chicken populations from geographically close countries such as Vietnam and Thailand shared the same genetic background at K = 5. Breeds from Finland, Saudi Arabia, Egypt, Switzerland, Turkey, the Netherlands, and Vietnam also shared a genetic background.

### Biodiversity across countries

**Fig. 4:**
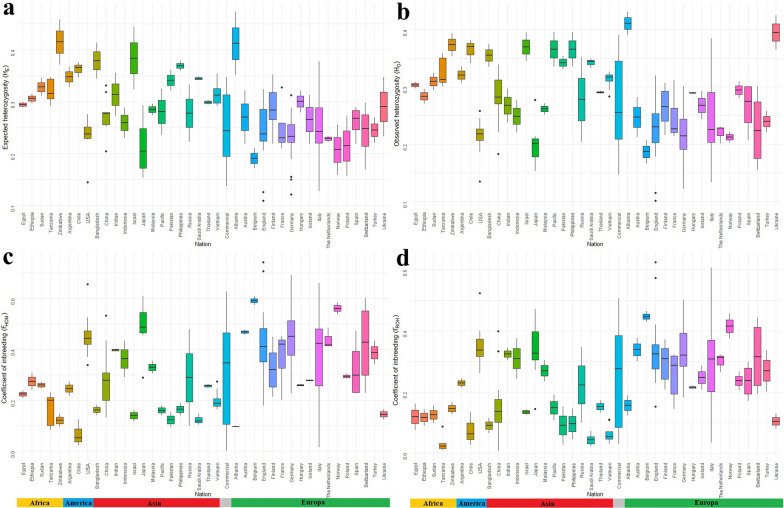
**a** Expected heterozygosity, **b** Observed heterozygosity, **c** Coefficient of inbreeding due to excess of homozygosity, and **d** Estimated homozygosity coefficient based on runs of homozygosity for each chicken breed group by country of origin

Figure 4 depicts boxplots of estimated indices for genetic diversity by geographical area. The upper part focuses on H_E_ and H_O_ (Fig. 4, b), while the bottom depicts F_HOM_ (Fig. 4c) and F_ROH_ (Fig. 4d), thus providing insights into the level of chicken genetic diversity within each country, as estimated based on the breeds represented in our dataset. High levels of average heterozygosity were observed in breeds from African regions such as Egypt, Ethiopia, Sudan, Tanzania, and Zimbabwe. Additionally, breeds from Bangladesh, Israel, and Albania exhibited considerable average heterozygosity (Fig. 4, b). Lower average heterozygosity was estimated for breeds originating from Belgium, while Italy reported moderate heterozygosity, albeit with a high variation within Italian breeds, resulting in a notably high standard deviation. Commercial lines, as expected, had the highest degree of genetic variability. The alignment of H_E_ and H_O_ suggests that the populations did not deviate from Hardy–Weinberg equilibrium.

The findings in Fig. 4c, d align with those of H_O_ and H_E_. Specifically, Fig. 4c shows that the coefficient of inbreeding was notably low in countries with high heterozygosity levels. The F_HOM_ was particularly high in breeds of Japanese, Belgian, and Norwegian origin. Italian breeds exhibited considerable variability for the F_HOM_ index, placing Italian chicken diversity at an average level compared to other countries. Commercial lines had a values close to the Italian ones.

Figure 4d reports the F_ROH_ and results mirror those obtained in Fig. 4c, with Belgian, Japanese, and Norwegian breeds having high inbreeding. Similarly, breeds from Italy and commercial lines exhibited broad variability, resulting in a slightly higher-than-average inbreeding value. It is noteworthy that African breeds, like those from Egypt, Ethiopia, Sudan, Tanzania, Zimbabwe, had lower inbreeding than breeds from other countries (in Fig. 4c, d).

### Population structure of Italian chicken breeds

**Fig. 5:**
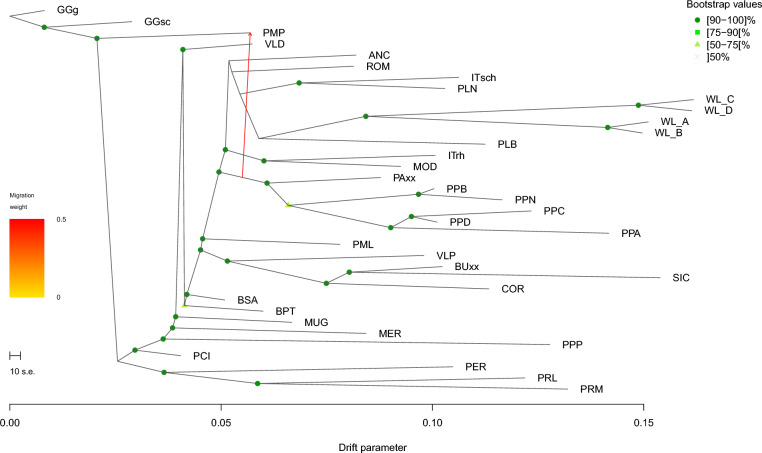
Maximum likelihood phylogenetic tree inferred using TreeMix with one migration edge

The TreeMix analysis was conducted for Italian chicken breeds as well as some Italian breeds not included in our dataset but obtained from the dataset of the SYNBREED project (ITrh, ITsch, PAxx and BUxx), to assess data congruence. Four commercial White Leghorn (WL) lines that are known for their similarity to the local breeds PLB and PLN were also included. The results suggest an evolutionary model in which several populations have experienced significant migration events. Populations such as WL_A, WL_B, WL_C, WL_D, ITsch, and MOD exhibited varying degrees of genetic drift and complex migratory flows. Phylogeographic clustering was observed on a regional scale among the chicken breeds. WL_A, WL_B, WL_C, and WL_D clearly derived from the two Leghorn populations (PLN and PLB), while the ITsch breed (Italian origin but raised in Germany) showed a close relationship with PLN (Fig. 5, see Additional file 2: Figure S2 and Additional file 3: Figure S3). Additionally, the ITsch breed separated from the closely related MOD due to genetic drift. A migration event from the branch of Padovana breeds (PPA, PPD, and PPC) to PMP was also evident, indicating an introgression from a common ancestor that PMP shares with these Italian breeds.

## Discussion

The TuBAvI and TuBAvI-2 projects fully aligned with the National Rural Development Program (PSRN) Biodiversity, which aimed to support conservation of animal genetic resources of zootechnical interest and to maintain genetic variability. To understand the importance of Italian chicken breeds and contextualize the local genetic heritage internationally, the dataset of Malomane et al. [8] was used. An important factor limiting large scale comparisons of genetic diversity across chicken breeds is often the heterogeneity of the molecular tools used (e.g., different SNP arrays, whole-genome sequencing, microsatellites), which prevents integration of the genotypic data. In the present study, all Italian breeds were genotyped for the same high-density SNP chip (600 K) as used by Malomane et al. [8], which ensured full integration of the datasets for the assessment of local chicken biodiversity within the international context. This highlights the importance of harmonizing genotyping strategies to support reliable and integrative cross-study evaluations.

The wealth of information available allowed us to investigate the effectiveness of conservation plans that were implemented in Italy for the protection of chicken biodiversity. It also enabled detailed study of the evolutionary and phylogenetic aspects of the Italian breeds. Additionally, we explored the level of variability within populations and conducted comparisons between breeds. Our analyses aimed to evaluate how the results of different diversity spectrum assessments aligned with our anticipated results. These expectations were formulated based on sampling locations, historical documentation, findings from previous studies, and our own understanding of the breeds'histories.

The imbalance in breed representation across countries within the dataset highlights the inherent challenges of assembling a truly global collection of chicken breeds. The limited representation of non-European breeds can be attributed to several factors, including the absence of centralized breeding facilities such as conservation centers in many countries. In less developed regions, chicken breeds are often maintained by small-scale private farmers for subsistence or local needs, which limits their accessibility for scientific studies. Furthermore, not all regions exhibit the same level of breed diversity as Europe. This disparity is shaped by a combination of socio-economic, political, and cultural factors that influence breed conservation and accessibility. These limitations reflect the difficulty of achieving complete global representation of chicken breeds within a single dataset. While the uneven distribution of breeds across countries could introduce biases into certain analyses, the overall robustness of the study remains unaffected. The dataset encompasses a wide range of breeds from diverse genetic, geographic, and phenotypic backgrounds, ensuring that the core patterns and insights are not disproportionately skewed by the overrepresentation of breeds from specific regions. Nevertheless, this study emphasizes the importance of future efforts to include breeds from less represented areas. Such endeavors would further enhance the comprehensiveness and global applicability of future analyses while reinforcing the significance of the present study as a foundation to increase the biodiversity of chicken populations.

### Italian chicken breeds and their relationships across countries

The MDS analysis (Fig. 1) provided a comprehensive view of the genetic relationships among the represented chicken breeds, with a particular focus on Italian breeds within the global chicken heritage. The Italian breeds were segregated from the rest of the dataset to assess their closest genetic cluster at the continental level. The Italian breeds shared significant genetic relationships with the majority of European and African breeds and, to a lesser extent, with Asian breeds. This finding was consistent with historical accounts of poultry trade and migration routes, which facilitated gene flow across these regions [23, 25]. The distinct isolated cluster that overlaps with commercial lines suggests a shared genetic background, potentially influenced by selective breeding practices aimed at enhancing certain commercial traits. However, we cannot exclude the possibility that this overlap may result from historical crossbreeding of local breeds with commercial lines [26, 27].

In detail, some Italian breeds had genetic connections with Austrian, Spanish, and German breeds, indicating historical gene flow and breeding exchanges between these countries (Fig. 1b) [10, 39, 40]. Additionally, certain Italian breeds clustered with breeds from Malaysia, Egypt, and Israel, suggesting more ancient or less direct genetic exchanges that were possibly facilitated through Mediterranean trade routes or the effect of colonialism, as for the other species [41, 42]. Berthouly et al. [43] observed that certain French and German chicken breeds exhibit genetic relatedness to African and Asian breeds, suggesting historical connections or shared ancestry.

To gain more detailed insights into the genetic proximity of Italian breeds to their global counterparts, Fig. 1c depicts the MDS plot for each Italian breed. Notably, Italian breeds such as PRL, PER, PCI, PRM, and ITrh overlapped significantly with commercial lines, indicating a strong genetic influence of the commercial breeding programs on the local ones. This overlap suggests that these Italian breeds were selectively bred to enhance traits favored in commercial production, such as egg laying or growth rate [44, 45]. Italian breeds that align with the European context, such as PPD, PPC, PPA, PPN, and PPB, were identified as more recent breeds in Italy. This aligns with their genetic proximity to other European breeds, suggesting recent breeding efforts to integrate desirable traits from Italian regions or vice versa [45]. Interestingly, southern Italian breeds such as SIC and BUxx showed genetic alignment with breeds from African countries, such as Egypt. This could reflect historical genetic exchanges through Mediterranean trade networks or adaptive traits selected for similar environmental conditions [46–48]. However, the SIC breed showed different background to the BUxx breed of Malomane’s dataset [8], although they should be the same breed. This could be attributed to the fact that the BUxx breed was raised in Germany, where it might have undergone crossbreeding with other native breeds, compromising the purity of its genetic background [8].

The distribution of Italian breeds within the phylogenetic tree (Fig. 2) was closely intertwined with various global breeds, indicating their genetic proximity to breeds from multiple countries. For example, the PCI breed showed close genetic alignment with both commercial lines and with the Hungarian YH breed, which was derived from the Hungarian TNN. This suggests a shared history of breeding purposes or gene flow, likely linked to the introduction of the TNN breed in Italy over 100 years ago to develop a new breed [14, 45, 49, 50]. However, the PCI breed has been shown to not share the same haplotype as TNN within region 104,754,409–105,526,289 on chromosome 3, which is of significant importance for the naked neck phenotype [51]. This may explain why PCI showed greater similarity to YH and was not directly associated with TNN. Similarly, the proximity of PPP and MER to the German DSgp breed suggests genetic exchanges or parallel selective breeding practices aimed at enhancing specific traits [27]. Southern Italian breeds, including VLP, COR, and SIC, formed a distinct branch in the phylogenetic tree, indicating a strong genetic association within this group. The BUxx breed also clustered closely with these breeds, reinforcing their shared genetic heritage. This branch of the phylogenetic tree reflects both their geographic and genetic uniqueness, likely shaped by regional breeding practices and environmental adaptations [24, 52]. On the other hand, breeds like PPA, PPC, PPD, PPN, and PPB formed a separate branch that somewhat connected to the Turkish-origin GZxx, suggesting historical connections between Italy and the Near East. Ceccobelli et al. [53] highlighted a significant genetic relationship between certain Italian local breeds and those from the Middle East and Asia based on mitochondrial DNA analyses. This connection may be attributed to historical events, such as the expansion of the Persian Empire and the Mongol invasions, which facilitated the movement of people and their livestock across regions, leading to genetic exchanges between European and Asian animal populations. The Mongol invasions, in particular, had a profound impact on the genetic makeup of various regions. Studies have shown that these invasions altered the genetic composition of populations in Eastern Europe, including Hungary, through the introduction of new genetic lineages [54]. Additionally, the expansion of the Mongol Empire established vast trade networks across Asia and Europe, promoting cultural and genetic exchanges. This period, known as the Pax Mongolica, allowed for the movement of goods, people, and animals, further contributing to the genetic intermingling of livestock breeds across continents [55].

The genetic diversity and relationships identified in the MDS and phylogenetic analyses has significant implications for the conservation and breeding of Italian chicken breeds. Cendron et al. (2020) [24] thoroughly investigated the Italian poultry heritage but they did not investigate relationships with breeds outside the national territory. The results of the current study provide a valuable starting point to enhance the knowledge of local poultry biodiversity. Understanding the genetic background of the various breeds could enable the recovery of breeds that are endangered and at risk of extinction [44, 56, 57]. Therefore, genetic revitalization of breeds that are preserved in conservation centers could be achieved by implementing crossbreeding programs with genetically similar external breeds. This approach, however, should be carefully balanced to ensure that the unique genetic characteristics of the local breeds are preserved, preventing the loss of specific traits that may be crucial for their identity and adaptation to local environments [25, 56].

### Comparing Italian biodiversity with countries worldwide

The ADMIXTURE analysis delineated the genetic landscape of global chicken breeds, with K = 25 providing the most comprehensive model for population differentiation. This underscores a notable degree of genetic distinctiveness among the breeds, reflecting a deep-seated genetic population structure shaped by both historical and contemporary breeding practices [25, 40, 58]. The clear genetic bifurcation at K = 2, which separated European breeds from those of other continents, aligned with historical geographical barriers and selective breeding practices [25, 40, 58, 59]. At K = 22, Italian breeds, such as PLN, PLB, and MOD were found to have genetic similarities with German and French breeds, which indicated historical gene flow and breeding exchanges facilitated by trade and movement of breeders across Europe [25, 58, 59]. This was consistent with historical accounts of the exchange of genetic material during significant agricultural developments in Europe [60, 61]. At K = 35, the close genetic relationship between the PMP breed and Swiss breeds (APsscht and APxx) suggested ongoing or recent gene flow between these populations [40, 62, 63].

The lack of genetic similarity between the EUK, HYL, ISA, and other commercial lines reported in Malomane et al. [8] emphasizes the distinct genetic selection criteria or their genetic drift that are employed in different commercial breeding programs [8]. Furthermore, the commercial breeds mentioned in Malomane et al. are described as'purebred white/brown layer lines,'which are probably the precursors of the current commercial hybrids included in our dataset. The close genetic resemblance of commercial line 708 to other broiler breeds (BRS_A, BRS_B, BRD_A, BRD_B) reflects a convergence in breeding objectives focused on meat production efficiency, consistent with other studies that have documented convergence in commercial breeding lines due to common selective pressure on specific production traits [27, 40].

In general, the observed genetic patterns highlight the influence of historical trade routes and animal movement on the genetic diversity of chicken breeds. The genetic similarities between Saudi Arabian and Egyptian breeds likely reflected historical trade routes that facilitated the exchange of poultry and breeding stock between these regions. Tixier-Boichard et al. [25] emphasized that historical trade and migration patterns have played a crucial role in shaping the genetic structure of poultry populations. Our findings were consistent with these observations, suggesting that trade and movement have led to significant gene flow and genetic admixture across regions [25].

Similarly, the genetic proximity observed between Vietnamese and Thai breeds could be attributed to their geographical proximity and shared agricultural practices [64–66]. Mtileni et al. [48] noted that such geographical and cultural factors often lead to genetic similarities among neighboring regions [48]. Our results supported this, indicating that shared agricultural practices and close geographic proximity have facilitated the exchange of genetic material between these Southeast Asian regions.

Analysis of genetic diversity indices (Fig. 4) revealed substantial differences in genetic diversity across regions. African breeds, including those from Egypt, Ethiopia, Sudan, Tanzania, and Zimbabwe, exhibited notably high levels of heterozygosity (Fig. 4a b). Specifically, the heterozygosity estimated for these regions, ranging from 0.29 to 0.42, were among the highest observed in our dataset. This suggests a rich genetic diversity, which may be attributed to the diverse breeding environments and lower selection pressures in these regions [48, 63]. Conversely, European breeds, such as those from Belgium, showed lower levels of heterozygosity, reflecting more pronounced genetic bottlenecks and selective pressures [40]. Italian breeds displayed moderate heterozygosity but considerable genetic variability among breeds, resulting in a high standard deviation of the diversity indices. This variability may be the result of distinct breeding practices and selective pressures within Italy, leading to differing levels of genetic diversity among Italian breeds [24, 27]. Such variability suggests not only considerable biodiversity but also substantial genetic variation within the Italian poultry populations.

Estimates of F_HOM_ (Fig. 4c) highlighted that breeds from regions with high heterozygosity generally show lower inbreeding coefficients. This is consistent with findings by Leroy et al. [67], who reported that high genetic diversity was often associated with lower levels of inbreeding [66]. In contrast, Japanese, Belgian, and Norwegian breeds exhibited high inbreeding coefficients, reflecting smaller effective population sizes in these regions [27, 68–71]. Italian breeds showed considerable differences in levels of inbreeding (0.38 ± 0.07), which places them at an intermediate level compared to other regions. This variability is indicative of the diverse breeding strategies employed in Italy. Estimates of F_ROH_ corroborated these findings, with Belgian, Japanese, and Norwegian breeds having pronounced levels of inbreeding. Italian breeds and commercial breeds also had notable inbreeding levels but larger differences in inbreeding, reflecting the different breeding practices and management strategies [27]. The reduced inbreeding levels observed in African breeds (e.g., Egypt = 0.076 ± 0.015; Ethiopia = 0.117 ± 0.102) compared to European and Japanese breeds suggest lower selection pressure in these regions. This finding supports the notion that regions with less intensive selection practices tend to maintain higher levels of genetic diversity and lower inbreeding [48].

The imbalance in the number of breeds from Italy compared to other countries in the studied dataset may, however, limit the direct comparisons across regions. Compared with other geographic areas, Italy has a rich biodiversity for chickens that is characterized by a large number of well-documented and conserved local breeds. This imbalance highlights a broader challenge in achieving equal representation of breeds globally, as the availability and documentation of chicken breeds varies significantly across regions due to differences in historical, socio-economic, and cultural contexts [39, 58].

### Genetic drift in Italian chicken breeds

The TreeMix analysis of Italian chicken breeds revealed significant insights into the genetic relationships and migration patterns among various breeds. TreeMix is a powerful tool for understanding the historical gene flow and genetic drift in populations by modeling the ancestry of populations and inferring migration events. This analysis was particularly relevant for Italian chicken breeds, given their rich history and diverse genetic backgrounds.

The tree topology identified by TreeMix indicated a well-resolved genetic structure among the sampled populations. The drift parameter, shown along the branches, reflects genetic divergence from the ancestral population. The breeds PLB, PLN, and ITsch clustered closely together, suggesting a shared genetic history and minimal genetic drift among them. This clustering was consistent with previous studies that have highlighted the genetic homogeneity of Italian breeds due to historical breeding practices aimed at maintaining certain phenotypic traits [24, 55, 72]. In contrast, breeds such as VLD and BSA, which were further apart on the tree, exhibited higher drift parameters, indicating greater genetic differentiation, which could be the result of geographical isolation, distinct breeding practices, or historical admixture events that introduced new genetic material into these populations [40].

One of the significant features of the TreeMix analysis was the identification of migration events. These are depicted by the arrows on the tree (Fig. 5), representing gene flow between populations, with the weight of the arrows indicating the proportion of ancestry derived from the migration event. For instance, the migration arrow from the Padovana breeds (PPN, PPB, PPC, PPD) to PMP suggests substantial gene flow from these breeds into PMP, highlighting historical introgression events that have shaped the genetic makeup of this population. Such gene flow events are crucial for maintaining genetic diversity and adaptive potential in breeds. They can introduce beneficial alleles that enhance disease resistance, productivity, or environmental adaptability [44]. The gene flow between Padovana breeds and PMP may have been facilitated by historical trade routes or deliberate crossbreeding efforts to improve and reconstruct an endangered breed like PMP [24, 44].

The genetic diversity within and between the Italian chicken breeds has significant implications for their conservation and management. The clustering of breeds with minimal drift, such as PLB, PLN, and ITsch, suggests that these populations have been relatively stable and maintained through controlled breeding practices. However, the high drift parameters observed in breeds like VLD and BSA indicate a need for conservation efforts to preserve their unique genetic heritage.

The identification of migration events also highlights the dynamic nature of genetic diversity in these populations. Conservation strategies should consider the historical gene flow and potential for future genetic exchange to maintain or enhance genetic diversity. This is particularly important in the context of climate change and emerging diseases, where genetic diversity can provide the necessary resilience for populations to adapt to changing conditions [44, 73].

The findings from the TreeMix analysis were consistent with other genomic studies on poultry breeds. For instance, Mtileni et al. [46] reported significant genetic diversity and historical gene flow in African chicken populations, which parallels the gene flow observed in Italian breeds [48]. Similarly, Groeneveld et al. [39] highlighted the role of historical trade routes in shaping the genetic diversity of livestock populations, which is evident in the migration events identified in the TreeMix analysis [39]. Moreover, the clustering of Italian breeds with minimal drift aligned with the findings of Liu et al. [23], who reported genetic homogeneity within European poultry breeds due to historical breeding practices. These comparisons underscore the reliability of the TreeMix analysis and its utility in uncovering the genetic history of poultry breeds.

Future research should focus on integrating genomics with phenotypic and environmental data to understand the adaptive significance of the observed genetic patterns. Studies on the genetic basis of specific traits, such as disease resistance or productivity, can provide valuable insights for breeding programs aimed at improving these traits in Italian chicken breeds [70]. Additionally, exploring the impact of climate change on the genetic diversity of these populations can inform adaptive management practices that ensure the long-term sustainability of poultry breeds [44].

## Conclusion

The genetic analysis performed in this study provides new insights into the biodiversity of Italian chicken populations within the global genetic context and the importance of appropriately managing them to prevent genetic erosion. Results confirm the distinctive genetic composition of Italian chicken breeds and their position within the global poultry heritage. The genetic relationships between Italian breeds and those from various African and Asian regions reflect both shared ancestry and region-specific evolutionary paths. Population structure analyses further highlighted the genetic diversity within Italian breeds, revealing both their divergence and similarities with international breeds, thus offering valuable information on their historical and geographical origins. By integrating global comparisons, this study emphasizes the broader significance of conserving local biodiversity, not only within Italy but also as part of a worldwide effort to maintain genetic diversity and resilience in poultry populations.

## Supplementary Information

Additional file 1: Figure S1. Title: Shared SNPs and blocks between TNN and PCI

Additional file 2: Figure S2. Title: Heatmap representing the level of genetic drift among populations

Additional file 3: Figure S3. Title: Heatmap of residuals representing the discrepancies between the migration tree model and the observed data among populations

## Data Availability

The datasets generated and/or analyzed during the current study are not publicly available but are available from the corresponding author upon reasonable request.
