# Supplementary figures and images for: Italian local chicken breeds: a comparative analysis of biodiversity on a global scale
Source: Genet Sel Evol. 2025 Jun 10;57:30. doi: 10.1186/s12711-025-00980-4 (PMC12150453; doi:10.1186/s12711-025-00980-4)

## Drift

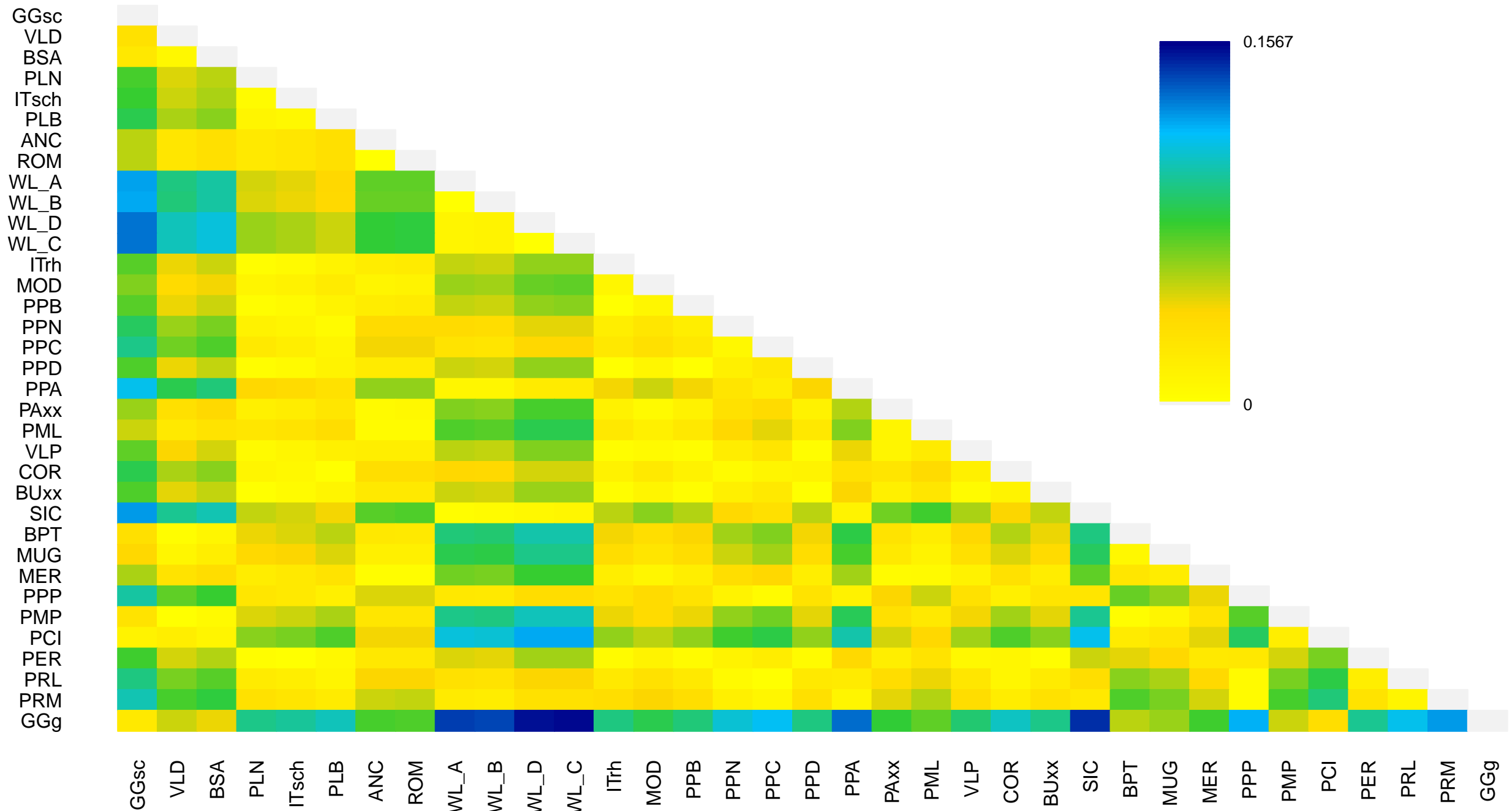

Supplement: Supplementary file 2 — Additional file 2: Figure S2. Title: Heatmap representing the level of genetic drift among populations [file 12711_2025_980_MOESM2_ESM.pdf]

# Residuals

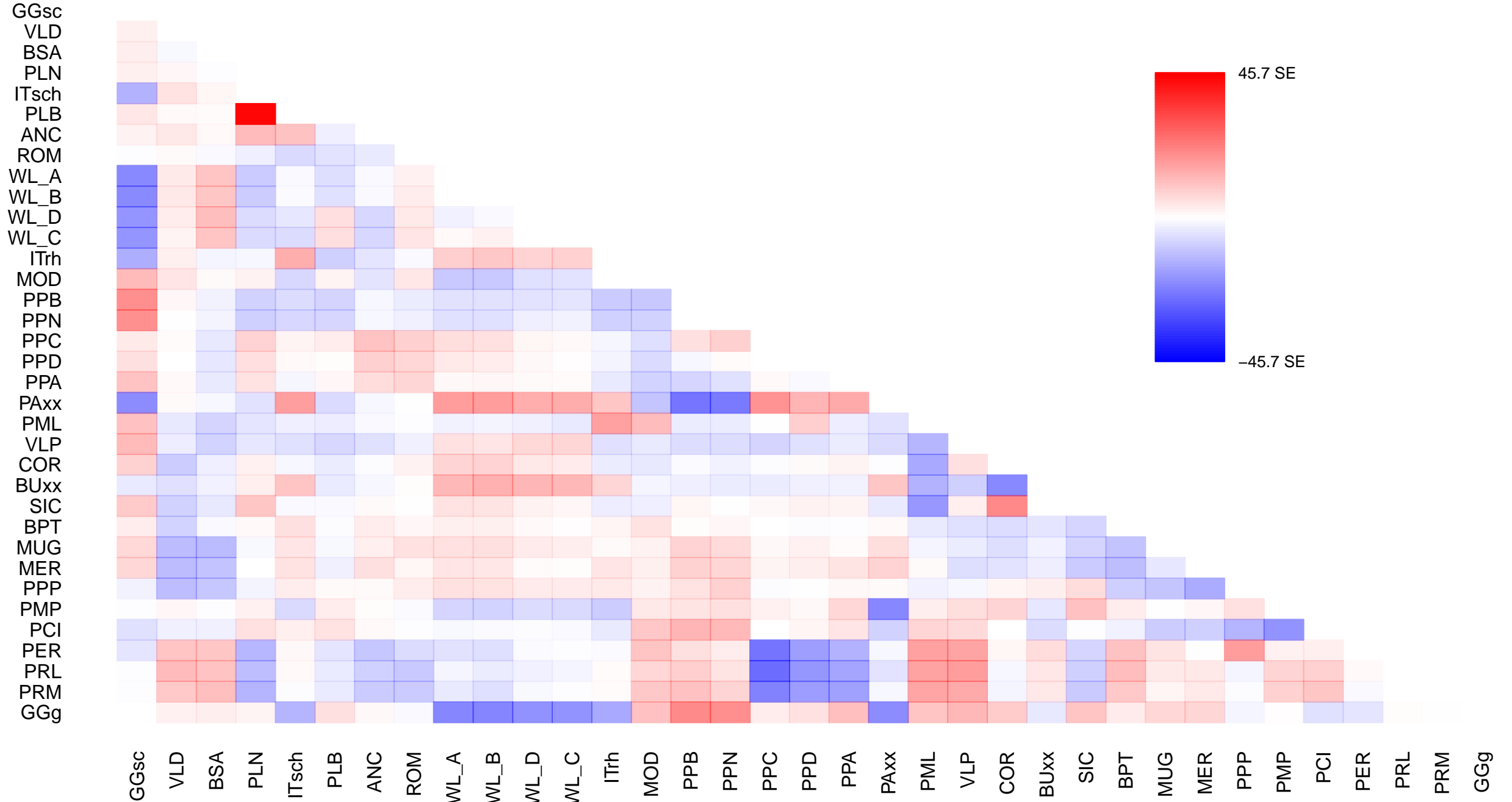

Supplement: Supplementary file 3 — Additional file 3: Figure S3. Title: Heatmap of residuals representing the discrepancies between the migration tree model and the observed data among populations [file 12711_2025_980_MOESM3_ESM.pdf]
